# Successful thoracoscopic enucleation of a calcifying fibrous tumor of the lower mediastinum in a young woman

**DOI:** 10.1186/s40792-024-01981-z

**Published:** 2024-08-01

**Authors:** Ryo Yokota, Takeshi Matsutani, Keisuke Mishima, Ryo Yamagiwa, Hirotoshi Kubokura, Norio Motoda, Nobuhiko Taniai, Hiroshi Yoshida

**Affiliations:** 1 Department of Digestive Surgery, Nippon Medical School Musashikosugi Hospital, 1-383, Kosugimachi Nakahara-Ku, Kawasaki-Shi, Kanagawa, 211-8533 Japan; 2 Department of Thoracic Surgery, Nippon Medical School Musashikosugi Hospital, Kanagawa, Japan; 3 Department of Diagnostic Pathology, Nippon Medical School Musashikosugi Hospital, Kanagawa, Japan; 4 Department of Gastrointestinal Hepato-Biliary-Pancreatic Surgery, Nippon Medical School Hospital, Tokyo, Japan

**Keywords:** Calcifying fibrous tumor, Pleura, Thoracoscopic tumor enucleation

## Abstract

**Background:**

Calcifying fibrous tumor (CFT) arising from the pleura is a relatively rare benign lesion in young and middle-aged adults. We report a 31-year-old woman with pleural CFT who underwent successful complete thoracoscopic enucleation.

**Case presentation:**

An asymptomatic woman presented with a mass in the right lower lung field that was incidentally detected on a chest X-ray during a routine medical checkup. Chest computed tomography showed a well-defined mass in the lower mediastinum, with a maximum diameter of approximately 5.5 cm. Esophagogastroduodenoscopy showed no abnormal findings in the esophagus. An endoscopic ultrasonography (EUS) revealed a well-defined tumor with no internal blood flow. EUS-fine needle aspiration failed to establish a definitive diagnosis. Therefore, thoracoscopic tumor enucleation was performed for diagnostic and therapeutic purposes. Based on the histopathological findings of the resected specimen, the presence of a tumor with a high fibrous component in a young woman, and the identification of granulomatous calcifications, a diagnosis of CFT was established.

**Conclusions:**

Complete thoracoscopic tumor enucleation was successfully performed for CFT arising from the pleura in a young adult woman.

## Introduction

Calcifying fibrous tumors (CFTs) are relatively rare benign lesions found that may occur as solitary or multiple lesions in any part of the body. These lesions have characteristic histologic and immunohistochemical features. Although most cases are diagnosed in childhood, CFTs may occur in young and middle-aged adults. The first pediatric case of CFT was reported by Rosenthal et al. in 1988 [1], and the disease definition was proposed by Fetsch et al. in 1993 [2]. CFTs arising from the pleura were first reported by Pinkard et al. in 1996 [3]. In recent years, the number of reported cases of CFTs has increased, predominantly involving the extremities and axillae. However, CFTs arising from the pleura are relatively rare. In this report, we describe our experience of CFT arising from the pleura in a young woman. The diagnosed was based on postoperative pathology after complete thoracoscopic enucleation. In addition, we briefly review the contemporary literature on pleural CFT cases.

## Case report

**Fig. 1:**
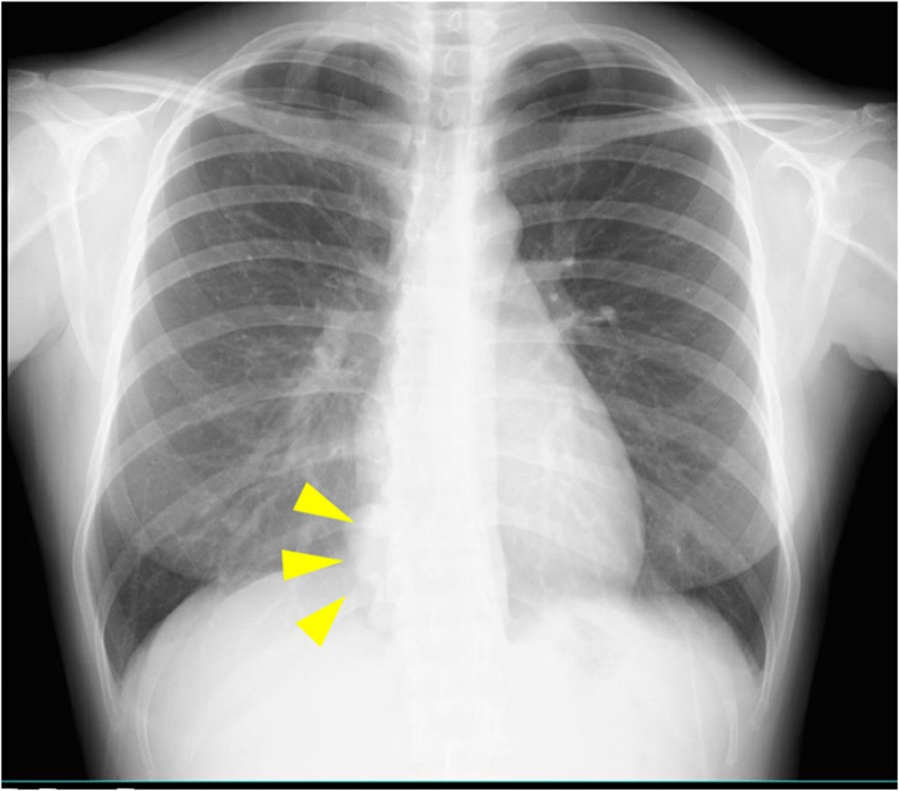
Chest X-ray showing a tumor in the right lower lung field (yellow arrows)

**Fig. 2:**
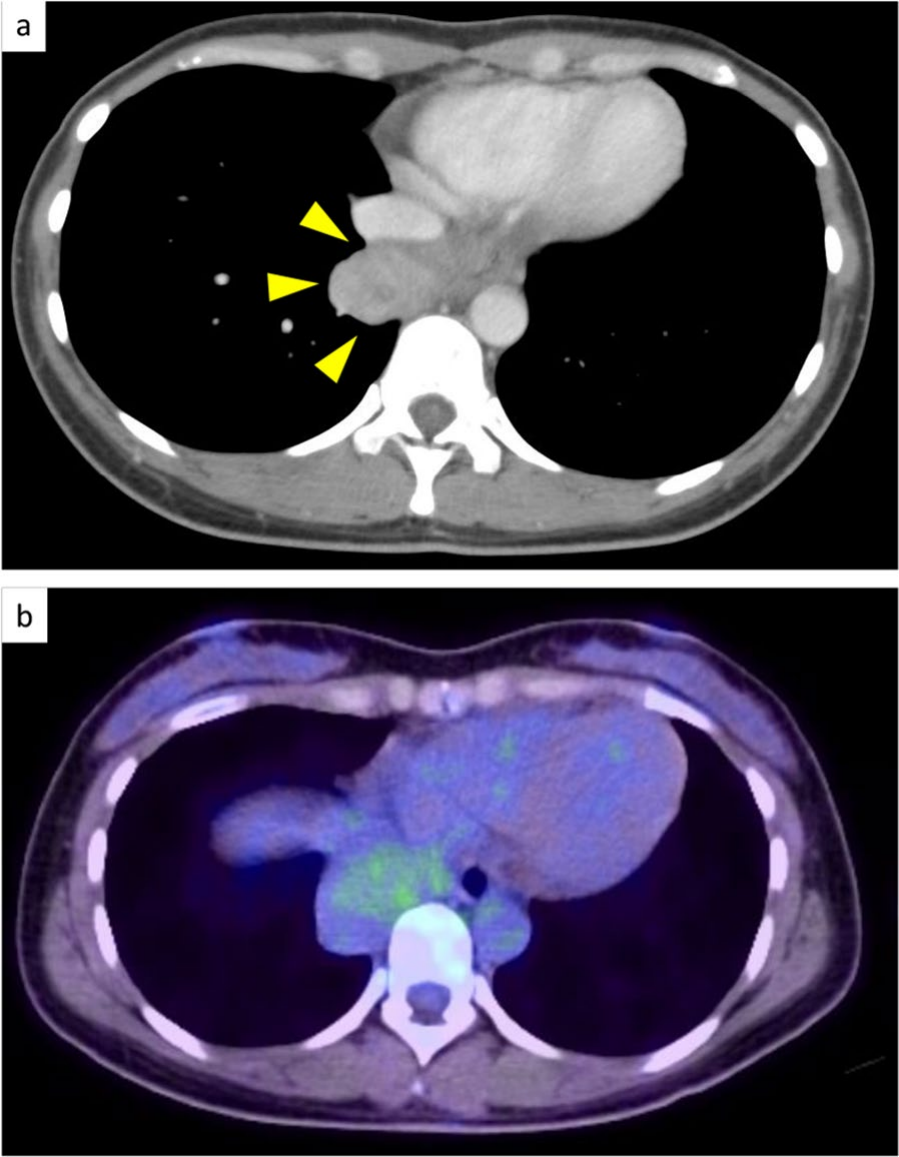
**a** CT image showing a large mass abutting the inferior vena cava, and esophagus (yellow arrows). **b** 18F-fluorodeoxyglucose positron emission tomography with CT (FDG-PET/CT) image showing FDG accumulation in the tumor with a maximum standardized uptake (SUVmax) of less than 2.0

**Fig. 3:**
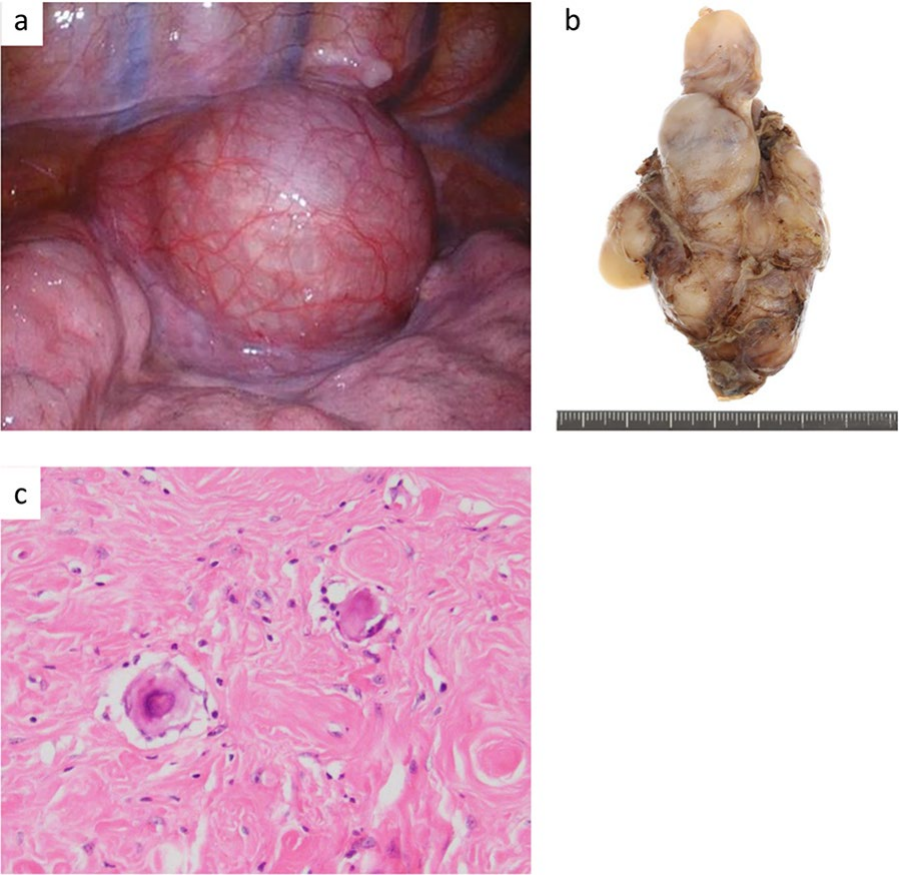
**a** Thoracoscopic intraoperative view of the large mass located in the lower mediastinum along the right side wall of the esophagus, the lung parenchyma, inferior vena cava, and the diaphragm. **b** Macroscopic findings of the tumor after complete surgical removal (size 85 × 55 × 20 mm). **c** Histopathological section showing bland spindle cells, densely hyalinized collagen, and psammomatous calcifications (hematoxylin and eosin stain 400 ×)

A 31-year-old woman was referred to our Department of Internal Medicine because of an incidentally detected mass in the right lower lung field on a chest X-ray performed as part of a routine medical checkup (Fig. 1). She had no physical symptoms and her medical history was unremarkable. Physical examination and laboratory findings were normal for her age. Serum levels of carcinoembryonic antigen and alpha-fetoprotein were within the normal range. Chest contrast computed tomography (CT) showed a tumor, approximately 5.5 cm in diameter, located near the posterior inferior vena cava and right side of the esophagus, with heterogeneous contrast enhancement in the delayed phase (Fig. 2a). 18F-fluorodeoxyglucose positron emission tomography with CT (FDG-PET/CT) imaging showed FDG accumulation in the tumor with a maximum standardized uptake (SUVmax) of less than 2.0 (Fig. 2b). CT-guided biopsy showed non-specific fibrous tissue, and did not lead to a definitive diagnosis by immunohistochemistry. Esophagogastroduodenoscopy revealed no abnormal findings in the lower thoracic esophagus. Endoscopic ultrasonography (EUS) revealed a well-defined tumor with no internal blood flow at a site 35 cm from the incisor teeth. As EUS-guided fine needle biopsy failed to confirm the diagnosis, in patients of this age and gender, the differential diagnoses include esophageal submucosal tumor, desmoid tumor, benign pleural tumor (solitary fibrous tumor, inflammatory myofibroblastic tumor), malignant tumor (desmoplastic malignant pleural mesothelioma), and pleural pseudotumor (calcified pleural plaques, chronic fibrous pleuritis, amylose, hyalinizing granuloma). Complete enucleation was performed via right video-assisted thoracoscopy in the prone position to obtain a definitive diagnosis. The tumor was located in the lower mediastinum along the right side wall of the esophagus and was in contact with the lung parenchyma, inferior vena cava, and the diaphragm (Fig. 3a). Complete enucleation of the tumor was performed. The tumor was a well-defined, multi-nodular enhancing lesion measuring 85 × 55 × 20 mm in size (Fig. 3b). Histopathological examination of hematoxylin and eosin-stained section revealed sparse spindle-shaped cells proliferation with mild atypia and prominent collagenous stroma with chronic inflammation; in addition, psammoma bodies were observed (Fig. 3c). On immunohistochemical staining, the spindle-shaped cells were focally positive for alpha-smooth muscle actin and negative for CD34, S-100, c-kit, DOG-1, STAT6, beta-catenin and ALK. Psammomatous calcification was observed in the paucicellular tumor with an abundant collagenous stroma, which led to the diagnosis of CFT. The postoperative course was uneventful, and the patient was discharged on postoperative day 8. There are no sign of recurrence as of 22 months of follow-up.

## Discussion

CFT is a relatively rare benign fibrous lesion that typically occurs in the soft tissues of the extremities, trunk, and neck, and rarely in the pleura. According to the bibliographic research of 157 cases by Chorti et al. in 2016 [4], the stomach (18%), small intestine (8.7%), and pleura (9.9%) are the most frequent sites of occurrence of CFT. The pathologic features of CFT are the proliferation of collagenous fibrous tissue with inflammatory cell infiltration and the presence of psammoma bodies. The etiology of CFT is not clear. However, CFT has been suggested to be an inflammatory pseudotumor caused by reactive changes involving osteopontin, a cytokine expressed during the repair process of inflammation. Osteopontin induces fibroblasts to form collagenous tissue and may also be involved in the formation of sand grain-like calcification [5]. As recently as 2002, the World Health Organization established the name “calcifying fibrous tumor” in its classification of soft tissue and bone tumors [6].

We conducted a literature search (January 1999 through December 2023) in the PubMed database and the Ichushi-Web database of the Japanese Medical Abstract Society (http://login.jamas.or.jp/; NPO Japan Medical Abstracts Society) using the keywords “calcifying fibrous tumor” and “pleura”. The 41 reported cases of CFT of pleural origin to date, including the present case, are summarized in Table 1 [3, 5–7]. The mean age of the 41 patients was 35 years (range, 15 weeks–59 years). None of the patients was aged > 65 years. CFT is more common in young females, with a male-to-female ratio of 14:27. Twenty-five patients had multiple lesions while 16 patients had single lesions. Eighteen patients had non-specific symptoms and 23 patients were asymptomatic. All 42 patients were divided into two groups: complete resection group (*n* = 28) and incomplete resection group (13 cases including one who did not undergo surgery), and their characteristics were compared and analyzed (Table 2). There were no significant differences in age (*p* = 0.576), gender (*p* = 0.386), or symptoms (*p* = 0.6324) between the two groups. Complete resection was possible in all 15 patients with solitary tumors, but the complete resection rate for multiple tumors was significantly lower (50%) compared to the solitary tumors. Multiple lesions extending the bilateral thoracic cavity or extensively throughout the entire region, both vertical or horizontal, were more likely to be unresectable, although not statistically significant. Reasons for incomplete resection on intraoperative findings included disseminated lesions in three cases, multifocal tumors in different sites in five cases, misdiagnosis in one case and not described in three cases; in one case, tumor resection was not chosen due to the presence of similar tumors in the thoracic and abdominal cavity on CT scan. There were no reports of tumor invasion making resection impossible. In the complete resection group, thoracoscopic surgery was twice as common as open thoracotomy, and the same ratio for the incomplete resection group. The median follow-up was shorter than 2 years in both groups, and no recurrence was reported in cases of complete resection group. On the other hand, in the incomplete resection group, one case of recurrence (details unknown) [7] and five cases of residual tumor were reported to have shown no growth.

**Table 1:** Reported cases of pleural calcifying fibrous pseudotumor

No.	Author	Year	Age	Gender	Focality	Symptoms	Surgery
1	Pinkard [3]	1996	23	F	Multiple	Present	Complete
2	Pinkard [3]	1996	28	F	Multiple	Absent	Complete
3	Pinkard [3]	1996	34	M	Solitary	Present	Complete
4	Hainaut	1996	29	F	Multiple	Absent	Incomplete
5	Cavazza	2002	46	F	Solitary	Absent	Complete
6	Ammar	2003	38	F	Solitary	Present	Complete
7	Jang	2004	31	F	Solitary	Absent	Complete
8	Soyer	2004	7	M	Solitary	Present	Complete
9	Mito	2005	54	M	Multiple	Absent	Incomplete
10	Kawhara	2005	35	F	Multiple	Present	Incomplete
11	Yasukawa	2006	35	F	Multiple	Present	Complete
12	Shibata	2008	52	F	Multiple	Absent	Incomplete
13	Suh	2008	35	M	Multiple	Absent	Complete
14	Miyano	2008	44	F	Multiple	Absent	Complete
15	Sleigh	2010	22	F	Multiple	Present	Incomplete
16	Yokosuka	2010	40	F	Solitary	Absent	Complete
17	Isaka [5]	2011	40	M	Multiple	Present	Complete
18	Jiang	2011	44	F	Multiple	Present	Complete
19	Agackiran	2012	40	M	Multiple	Present	Complete
20	Fujita	2012	58	F	Solitary	Absent	Complete
21	Ishida	2013	31	M	Multiple	Absent	Incomplete
22	Azam	2014	31	M	Multiple	Absent	No resection
23	Matsumoto	2014	20	F	Multiple	Absent	Complete
24	Nakagawa [6]	2014	30 s	F	Multiple	Present	Complete
25	Minerowic	2015	15	F	Multiple	Present	Incomplete
26	Lee	2015	47	F	Solitary	NA	Complete
27	Rocas	2015	59	M	Solitary	Absent	Complete
28	Sawaga	2017	55	F	Multiple	Absent	Incomplete
29	Edlin	2018	23	F	Solitary	Present	Complete
30	Mazi	2018	15	F	Multiple	Present	Complete
31	Lisowsk	2018	27	F	Solitary	Absent	Complete
32	Mehrad [7]	2018	32	M	Multiple	Present	Incomplete
33	Mehrad [7]	2018	21	M	Solitary	Absent	Complete
34	Mehrad [7]	2018	32	F	Multiple	Absent	Complete
35	Massoth	2019	59	M	Multiple	Absent	Incomplete
36	Bono	2020	10	M	Solitary	Present	Complete
37	Miyamoto	2020	21	F	Multiple	Absent	Incomplete
38	Gorai	2020	52	F	Solitary	Absent	Complete
39	Hernandez	2021	35	M	Multiple	Present	Complete
40	Jia	2021	38	M	Multiple	Present	Incomplete
41	Our case		31	F	Solitary	Absent	Complete

**Table 2:** Characteristics of patients with complete or incomplete resection of pleural calcifying fibrous pseudotumor

	Complete resection	Incomplete resection	*p*-value
All cases	28	13	
Age at diagnosis of tumor (years)			0.5763
Median	34	36	
Range	7–59	15–59	
Gender (male/female)	9/19	6/7	0.38616
Symptoms			0.6324
Present	13	5	
Absent	15	8	
Number of tumors			0.0009
Solitary	15	0	
Multiple	13	13	
Location of the lung field of the tumors			0.0547
Right	18	6	
Left	9	3	
Right + left	1	4	
Vertical location of the tumors			0.0853
Upper	1	0	
Upper + middle	0	1	
Middle	1	0	
Middle + lower	3	2	
Lower	20	5	
Upper + middle + lower	3	5	
Horizonal location of the tumors			0.0599
Central	6	2	
Lateral	15	3	
Central + lateral	3	6	
NA	4	1	
Operative methods			0.5
Open thoracotomy	7	2	
Thoracoscopic surgery	17	7	
No surgery	0	1	
NA	4	3	
Follow-up period (months)			0.1725
Midian	17	10	
Range	6–17	3–24	

*NA* not available

The differential diagnosis of pleural CFTs includes inflammatory myofibroblastic tumors, solitary fibrous tumors [8], sarcomatoid and desmoplastic mesothelioma, desmoid fibromatosis, leiomyoma, chronic fibrous pleuritis, and IgG4-related sclerosing disease. Immunostaining is useful in the differential diagnosis of these diseases. However, the lack of specific findings in CFT makes preoperative diagnosis difficult, even with CT-guided or endoscopic ultrasound needle biopsy. Furthermore, because of the difficulty in differentiating these diseases by imaging, complete resection for definitive diagnosis should be performed in cases with tumor enlargement and subjective symptoms. Mehrad et al. [7] recently reported deleterious mutations in three genes, Zinc Finger Protein 717 gene (*ZNF717*), Facioscapulohumeral muscular dystrophy-1 gene (*FRG1*), and cell division cycle 27 (*CDC27*), and abnormal copy number loss in chromosomes 8 and 6 by whole genome sequencing in CFT patients, suggesting that these molecular-level changes may contribute to the genesis of CFTs. Although the molecular profile of previously reported CFT cases has been examined to identify possible driver mutations, this patient did not consent to genetic testing.

The patient had no symptoms and the tumor was incidentally detected on chest X-ray. Preoperative CT revealed that the tumor was in contact with the esophagus, and we initially suspected an esophageal submucosal tumor. Intraoperative examination showed that the tumor was located on the right side of the esophagus and was broad-based. Owing to the suspicion of a mediastinal tumor, complete tumor resection was performed under thoracoscopy. Twenty-two months after surgery, the patient is being followed up without recurrence. However, there is no established interval or duration of follow-up for completely resected CFTs of pleural origin.

## Conclusion

We report a rare case of CFT originating from the pleura in a young adult woman. The etiology and prognosis of pleural CFT are not clear. Further accumulation of cases is needed in the future.

## Data Availability

The data are not available for public access due to patient privacy concerns but are available from the corresponding author on reasonable request.

## Abbreviations

CFT: Calcifying fibrous tumor
CT: Computed tomography
FDG-PET: Fluorodeoxyglucose positron emission tomography
EUS: Endoscopic ultrasonography
